# Age-Dependent Flowering Transition in *Cymbidium goeringii:* Physiological Characterization and Small RNA Profiling

**DOI:** 10.3390/plants15192899

**Published:** 2026-09-22

**Authors:** Zihan Xu, Tao Huang, Yue Chen, Changsheng Shao, Huijun Zheng, Chunjing Zou, Mingli Zhang, Luying Huang, Zhongxing Gong, Fengrong Hu

**Affiliations:** 1 Institute of Modern Service and Management, Hangzhou Polytechnic University, Hangzhou 310018, China; 2 College of Landscape Architecture, Nanjing Forestry University, Nanjing 210037, China; 3 Institute of Horticulture, Zhejiang Academy of Agricultural Science, Hangzhou 310021, China

**Keywords:** *Cymbidium goeringii*, age-dependent flowering, small RNA sequencing, miRNA, physiological characterization

## Abstract

Age-dependent flowering is a prominent feature of perennial orchids, yet its physiological and small RNA basis remains poorly understood in *Cymbidium goeringii*. Here, floral bud formation, carbohydrate contents, and endogenous hormones were examined across successive shoot age stages, and four representative stages were selected for small RNA sequencing. Floral bud formation showed a clear age-dependent rise-and-fall pattern, accompanied by distinct changes in carbohydrate and hormone levels. Small RNA sequencing identified 810 miRNAs, including 356 known and 454 putative novel miRNAs, of which 311 were differentially expressed. Temporal expression analysis grouped 210 DEMIs into four significant profiles, all showing reduced expression in the oldest shoots. Functional analysis of predicted targets highlighted starch and sucrose metabolism and plant hormone signal transduction, and further pathway analysis identified multiple predicted miRNA–target associations related to carbohydrate metabolism and hormone signaling. qRT-PCR analysis generally supported the sequencing-based expression patterns. These findings reveal coordinated age-related changes in flowering competence, physiological status, and miRNA expression, and identify candidate miRNAs and functional pathways potentially associated with age-dependent flowering in *C. goeringii*.

## 1. Introduction

*Cymbidium goeringii* is an important perennial ornamental species of the genus *Cymbidium*, with considerable horticultural and germplasm value [1]. Compared with annual plants, perennials undergo longer and more complex developmental programs, during which vegetative growth, the acquisition of reproductive competence, and subsequent developmental states may all be progressively influenced by age. Recent studies in perennial species such as grapevine and poplar have further shown that plant age is not merely a measure of chronological time, but is closely associated with shoot and meristem status, vegetative phase transition, and seasonal growth behavior [2,3]. *C. goeringii* has a prolonged vegetative phase and continuously produces shoots representing successive age stages. However, previous studies on flowering in *Cymbidium* have largely focused on morphological differentiation, physiological changes, and flowering regulation within a single floral bud differentiation cycle [4,5,6]. Consequently, how flowering competence changes in different age stages, and the physiological and molecular basis underlying this age dependence, remain insufficiently understood.

Age-related developmental transitions in plants are closely associated with internal metabolic and hormonal states [7]. Carbohydrates not only provide carbon and energy for vegetative growth and reproductive development but also function as signaling molecules in the regulation of plant developmental processes [8,9]. Recent evidence has further shown that sugar signaling can modulate the accumulation and activity of the key shoot apical meristem regulator SHOOT MERISTEMLESS, supporting a direct link between carbon status and meristem development [10]. In parallel, multiple phytohormones, including auxin, cytokinin, gibberellin (GA), abscisic acid (ABA), brassinosteroids (BRs), and jasmonates (JAs), participate in vegetative growth, developmental phase transitions, and flowering [11]. Crosstalk between hormonal signaling and age-related developmental pathways has also been reported, further highlighting the complex relationship between developmental age and hormonal regulation [12]. In *Cymbidium*, floral bud differentiation is accompanied by substantial changes in carbohydrate and endogenous hormone levels [4,5,6]. However, previous studies have primarily compared successive stages within a single floral bud differentiation cycle, and how these physiological traits vary with shoot age and relate to flowering competence remains largely unresolved.

miRNAs are an important class of endogenous non-coding small RNAs that regulate gene expression primarily through target mRNA cleavage or translational repression, and they are widely involved in plant growth, developmental transitions, hormone responses, and reproductive development [13,14]. Several conserved miRNA families, including miR156, miR159, miR167, miR172, miR319, and miR396, have been implicated in developmental timing, flowering, and reproductive organ development [15,16,17,18], indicating that age-related development is likely coordinated by multiple miRNA regulatory modules rather than by a single factor. With the development of high-throughput small RNA sequencing, numerous known and novel miRNAs have been identified in orchids, including *Phalaenopsis*, *Dendrobium*, and *Cymbidium* species [19,20,21,22,23]. In *C. goeringii* and other *Cymbidium* species, previous studies have mainly focused on floral morphology and floral organ development, identifying potential regulatory relationships involving miR156–SPL, miR319–TCP, miR396–GRF, and miR390-related modules [24,25,26]. However, existing small RNA studies in *Cymbidium* have largely centered on floral traits, specific developmental stages, or individual miRNAs. The global dynamics of miRNA expression across successive shoot age stages, as well as their potential links with concurrent metabolic and hormonal changes, have yet to be systematically characterized.

Accordingly, *C. goeringii* ‘Songmei’ was used to investigate age-dependent flowering and its associated physiological and miRNA changes. Floral bud formation was evaluated across six successive shoot age stages, together with measurements of major carbohydrates and endogenous hormones in leaves to characterize age-related physiological variation. Based on these patterns, four representative age stages were selected for small RNA sequencing. miRNAs and differentially expressed miRNAs (DEMIs) were identified, followed by analyses of their temporal expression patterns and the functions of their predicted targets. GO and KEGG analyses were further used to examine predicted miRNA–target associations related to starch and sucrose metabolism and plant hormone signal transduction, and selected miRNAs were validated by qRT-PCR. This study integrates age-related flowering phenotypes, physiological traits, and small RNA profiles to provide a basis for understanding age-dependent flowering in *C. goeringii* and to identify candidate miRNAs and functional pathways potentially associated with this developmental process.

## 2. Materials and Methods

### 2.1. Plant Materials

*Cymbidium goeringii* ‘Songmei’ was used as the experimental material. Self-pollination was performed in spring 2011, and nearly mature capsules were harvested in September of the same year and stored at 4 °C for 7 d. The seeds were subsequently aseptically sown on artificial culture medium and cultured in darkness to promote germination. Seed germination began in spring 2012, followed by rhizome proliferation, plantlet regeneration, and rooting. Beginning in autumn 2013, the regenerated plantlets were gradually transferred from in vitro culture and acclimatized in a multi-span greenhouse at the Institute of Horticulture, Zhejiang Academy of Agricultural Science, Hangzhou, China. After successful transplantation, the plants were maintained under natural light at approximately 70% relative humidity and 22 °C.

The plants began to bloom in spring 2018 and thereafter produced new shoots annually. In this study, shoot age refers to the chronological age of an individual shoot/pseudobulb, defined according to the year in which the shoot was produced. For the experiment conducted in 2023, shoots produced in the current year were designated Y0, whereas one- to five-year-old shoots were designated Y1–Y5, respectively. Shoots produced in successive years remained connected and were cultivated together in the same pot, and floral buds developed at the bases of the newly formed pseudobulbs.

### 2.2. Determination of Floral Bud Formation and Physiological Traits

#### 2.2.1. Floral Bud Formation Rate

Floral bud formation was evaluated in October–November 2023, when floral buds had become morphologically distinguishable at the bases of the pseudobulbs. 50 pots containing Y0–Y5 shoots were randomly selected for evaluation. For each age stage, 10 shoots were used as one biological replicate, with five biological replicates. The presence or absence of floral buds at the base of each pseudobulb was recorded, and the floral bud formation rate was calculated as the percentage of shoots bearing floral buds relative to the total number of shoots examined.

#### 2.2.2. Determination of Carbohydrate Contents

For physiological measurements, fully expanded mature leaves located in the middle portion of each shoot were collected from Y0–Y5 shoots at the early stage of floral bud formation. Tissue from the middle region of each leaf blade was sampled, avoiding the midrib and leaf margins. Leaves from 10 shoots were pooled as one biological replicate, with three biological replicates for each age stage. Samples were collected between 10:00 and 11:00 a.m., immediately placed on ice, and stored at −80 °C until analysis.

Soluble sugar and sucrose contents were determined using a modified anthrone–sulfuric acid method based on previously described procedures [27]. Fresh leaf tissue (0.2 g) was extracted three times with 80% (*v*/*v*) ethanol (prepared from absolute ethanol supplied by Sinopharm Chemical Reagent Co., Ltd., Shanghai, China) at 80 °C for 30 min, and the combined supernatants were adjusted to a final volume of 25 mL. For soluble sugar determination, an aliquot of the extract was directly reacted with anthrone–sulfuric acid (prepared using anthrone and sulfuric acid from Sinopharm Chemical Reagent Co., Ltd., Shanghai, China) reagent and heated in a boiling-water bath for 10 min. For sucrose determination, the extract was first treated with 30% KOH (prepared using potassium hydroxide from Sinopharm Chemical Reagent Co., Ltd., Shanghai, China) and heated in a boiling-water bath for 10 min before reaction with anthrone–sulfuric acid reagent. Absorbance was measured at 625 nm, and the contents were calculated using the corresponding standard curves.

Glucose content was determined using a glucose assay kit (BC2500, Solarbio, Beijing, China). Fresh leaf tissue (0.1 g) was extracted in distilled water, and the resulting supernatant was subjected to the enzymatic colorimetric reaction according to the manufacturer’s instructions. Absorbance was measured at 505 nm.

Starch content was determined using a plant starch assay kit (A148-1-1, Nanjing Jiancheng Bioengineering Institute, Nanjing, China). Fresh leaf tissue (0.1 g) was extracted and gelatinized according to the manufacturer’s protocol, followed by colorimetric determination at 620 nm. All carbohydrate contents were expressed on a fresh-weight basis.

#### 2.2.3. Determination of Endogenous Hormone Contents

The contents of indole-3-acetic acid (IAA), zeatin riboside (ZR), abscisic acid (ABA), gibberellic acid (GA), methyl jasmonate (MeJA), and brassinosteroids (BR) were determined using enzyme-linked immunosorbent assay (ELISA) as previously described [28]. Fresh leaf tissue (0.3 g) was extracted with 80% methanol containing 1 mmol L^−1^ butylated hydroxytoluene (both reagents supplied by Sinopharm Chemical Reagent Co., Ltd., Shanghai, China) at 4 °C for 4 h. After centrifugation, the supernatant was purified using C18 solid-phase extraction cartridges, evaporated to dryness, and redissolved in sample dilution buffer. Hormone concentrations were determined by competitive ELISA using monoclonal antibodies supplied by the Plant Hormone Research Institute, China Agricultural University, Beijing, China. Absorbance was measured at 490 nm, and hormone contents were calculated according to the method of Weiler et al. [28].

### 2.3. Small RNA Library Construction and Sequencing

Based on age-dependent differences in floral bud formation and physiological characteristics, Y0, Y1, Y3, and Y5 were selected as representative age stages for small RNA sequencing. Healthy leaves at the same position were collected from each age stage, with three biological replicates per stage. Samples were immediately frozen in liquid nitrogen and stored at −80 °C until RNA extraction. In total, 12 small RNA libraries were constructed.

Total RNA was extracted using TRIzol reagent (Invitrogen, Thermo Fisher Scientific, Waltham, MA, USA). Small RNA fragments of 18–30 nt were isolated by electrophoresis on a 15% denaturing polyacrylamide gel (prepared using acrylamide/bis-acrylamide from Sigma-Aldrich, St. Louis, MO, USA) containing 7 M urea (Sigma-Aldrich, St. Louis, MO, USA) in 1× TBE buffer. The gel was pre-run at 200 V for 15 min, followed by sample loading and electrophoresis at 200 V for approximately 60 min. The gel region corresponding to 18–30 nt was identified using a small RNA marker (New England Biolabs, Ipswich, MA, USA) and excised for RNA recovery. The recovered small RNAs were sequentially ligated to 3′ and 5′ adapters. The adapter-ligated RNAs were reverse-transcribed and PCR-amplified, and products of approximately 140 bp were recovered and purified for library construction. Library fragment-size distributions were assessed using an Agilent 2100 Bioanalyzer (Agilent Technologies, Santa Clara, CA, USA), and library concentrations were determined by qPCR before sequencing. Biotechnology Co., Ltd. (Guangzhou, China).

### 2.4. Small RNA Data Processing and miRNA Identification

Raw reads were filtered using fastp to remove reads containing more than one base with a quality score below 20 or any ambiguous bases (N), reads lacking a 3′ adapter or containing a 5′ adapter, reads with no insert or an insert shorter than 18 nt, and reads with an adenine content greater than 70%. Following 3′ adapter trimming, the retained sequences were used as clean tags for subsequent analyses. Small RNA tags were annotated against the GenBank and Rfam databases, and sequences corresponding to rRNAs, scRNAs, snoRNAs, snRNAs, and tRNAs were removed using BLASTALL v2.2.25. The remaining tags were mapped to the chromosome-level reference genome of *Cymbidium ensifolium* obtained from the Genome Warehouse (GWH) of the National Genomics Data Center, China National Center for Bioinformation (CNCB-NGDC; accession no. GWHBCII00000000).

Genome-mapped small RNA tags were compared with known plant miRNAs deposited in miRBase to identify known miRNAs. After removal of known miRNAs and other annotated small RNAs, putative novel miRNAs were predicted using MIREAP v0.2 based on precursor hairpin structures, Dicer cleavage characteristics, and minimum free-energy features. The length distribution of small RNA tags in each library was analyzed using the Omicsmart platform (https://www.omicsmart.com). Nucleotide preferences at individual sequence positions and at the 5′ terminal position across different length classes were analyzed separately for known and putative novel miRNAs on the same platform.

### 2.5. miRNA Expression and Differential Expression Analyses

The abundance of each miRNA was normalized as transcripts per million (TPM). Differentially expressed miRNAs (DEMIs) were identified using edgeR (v.4) with a fold-change threshold of ≥1.5 and *p* < 0.05. Six pairwise comparisons were performed: Y1 versus Y0, Y3 versus Y0, Y5 versus Y0, Y3 versus Y1, Y5 versus Y1, and Y5 versus Y3. Hierarchical clustering and heatmap visualization of the DEMIs were performed on the Omicsmart platform using their TPM expression profiles. In addition, the 20 DEMIs with the highest mean TPM values across Y0, Y1, Y3, and Y5 were selected to characterize highly abundant miRNAs.

Temporal trend analysis was further performed for the DEMIs across the four age stages. MiRNAs with similar expression trajectories were grouped into 20 profiles, and significantly enriched expression profiles were identified based on their corresponding *p*-values.

### 2.6. Target Prediction and Functional Analysis

Target genes of the identified DEMIs were predicted based on sequence complementarity between miRNAs and candidate target transcripts. The predicted targets were annotated using the Gene Ontology (GO) and Kyoto Encyclopedia of Genes and Genomes (KEGG) databases. GO enrichment analysis was performed using a hypergeometric test, and GO terms with adjusted *p* < 0.05 were considered significantly enriched. KEGG pathway enrichment analysis was similarly conducted using a hypergeometric test, and pathways with an FDR-adjusted *p*-value (*Q* value) ≤ 0.05 were considered significantly enriched.

For further functional interpretation, predicted targets associated with starch and sucrose metabolism and plant hormone signal transduction were extracted according to KEGG annotation. The corresponding predicted miRNA–target associations and miRNA expression patterns across Y0, Y1, Y3, and Y5 were mapped to the respective pathways.

### 2.7. qRT-PCR Validation

Stem-loop reverse transcription and qRT-PCR primers were designed using miRNA Design v1.01, and the primer sequences are listed in Supplementary Table S1. First-strand cDNA was synthesized from 1 μg of total RNA using the miRNA 1st Strand cDNA Synthesis Kit (by stem-loop) (MR101-01, Vazyme, Nanjing, China) according to the manufacturer’s instructions.

qRT-PCR was performed using TB Green^®^ Premix Ex Taq™ (RR420, Takara, Kyoto, Japan) in a 20 μL reaction volume. The amplification program consisted of an initial denaturation at 95 °C for 5 min, followed by 40 cycles of 95 °C for 5 s and 60 °C for 30 s, followed by melting-curve analysis. *U6* was used as the internal reference. Three biological replicates and three technical replicates were performed for each sample, and relative miRNA expression levels were calculated using the 2^−ΔΔCt^ method.

### 2.8. Statistical Analysis

Data from physiological measurements and qRT-PCR analyses were subjected to one-way analysis of variance (ANOVA) using SPSS v22.0 (IBM, Armonk, NY, USA). Data are presented as the mean ± SD of biological replicates. Differences were considered statistically significant at *p* < 0.05 and highly significant at *p* < 0.01.

## 3. Results

### 3.1. Age-Dependent Flowering Patterns and Physiological Characteristics of C. goeringii

#### 3.1.1. Floral Bud Formation in Different Age Stages

New shoots of *C. goeringii* ‘Songmei’ emerged between February and May following flowering and subsequently developed new pseudobulbs, at the bases of which floral buds formed. Shoots produced in successive years remained in the same pot and were classified according to their ages (Figure 1A). The current-year shoots were designated as Y0, whereas one- to five-year-old shoots were designated as Y1–Y5, respectively. No floral buds were observed in Y0 shoots. The floral bud formation rate was 18% in Y1, reached a maximum of 32% in Y2, and subsequently decreased to 12% in Y3. No floral buds were detected in Y4 or Y5 shoots (Figure 1B,C). Thus, floral bud formation initially increased and then declined with shoot age, with the highest formation rate observed in Y2 (Figure 1B,C).

#### 3.1.2. Age-Dependent Changes in Carbohydrate Contents

Carbohydrate contents in the leaves of *C. goeringii* ‘Songmei’ varied substantially among different age stages (Figure 2). Soluble sugar and sucrose contents increased markedly from Y0 to Y3 and subsequently remained at relatively high levels. Glucose exhibited the most pronounced variation, increasing continuously to a maximum in Y3, followed by a significant decrease in Y4 and a slight recovery in Y5. Starch content increased gradually with shoot age and reached its highest level in Y5. These results indicated that carbohydrate profiles differed among shoot age stages, reflecting age-related variation in carbon storage and utilization.

#### 3.1.3. Age-Dependent Changes in Endogenous Hormone Contents

Endogenous hormone contents also displayed distinct age-dependent patterns in the leaves of *C. goeringii* ‘Songmei’ (Figure 3). IAA, ABA, and BR generally increased with shoot age, with IAA and BR showing particularly pronounced changes. In contrast, MeJA declined progressively, while ZR decreased mainly from Y1 to Y3 and subsequently remained relatively stable. GA content varied only slightly from Y0 to Y4 but increased in Y5. Collectively, these contrasting trends indicated substantial remodeling of endogenous hormone balance in different age stages.

### 3.2. Overview of Small RNA Sequencing and Identification of miRNAs

Based on age-dependent differences in floral bud formation and physiological traits, four representative stages, Y0, Y1, Y3, and Y5, were selected for small RNA sequencing. A total of 12 libraries, comprising three biological replicates for each stage, were constructed and sequenced using the Illumina HiSeq 2500 platform (Illumina, Inc., San Diego, CA, USA). After quality filtering, 9.36–14.52 million clean reads and 8.56–13.13 million clean tags were obtained from each library. The small RNA sequences were predominantly 21 and 24 nt in length, with the 24-nt class showing the highest abundance (Figure 4A). The consistent length distributions and sufficient sequencing depth across the libraries supported their use for subsequent miRNA identification and expression analyses.

After removal of reads corresponding to rRNAs, tRNAs, snRNAs, snoRNAs, and other non-miRNA sequences, a total of 810 miRNAs were identified, including 356 known and 454 putative novel miRNAs. Among them, 549 miRNAs were detected in all four stages, whereas 59, 35, 46, and 22 miRNAs were specifically detected in Y0, Y1, Y3, and Y5, respectively (Figure 4B). Most known and putative novel miRNAs exhibited a preference for uracil at the 5′ terminus, whereas the 24-nt miRNAs preferentially began with adenine (Figure 4C–F). These results revealed a core set of miRNAs shared across developmental stages, together with a subset showing stage-specific expression.

### 3.3. Differentially Expressed miRNAs in Different Age Stages

A total of 311 unique differentially expressed miRNAs (DEMIs) were identified across the six pairwise comparisons. Among them, 113 miRNAs were upregulated, and 249 were downregulated in at least one comparison, while 51 miRNAs exhibited opposite expression trends among different comparisons. The largest numbers of DEMIs were detected in Y5 versus Y1 and Y5 versus Y0, with 162 and 157 DEMIs, respectively, most of which were downregulated. In contrast, only 22 DEMIs were identified between Y1 and Y3. Hierarchical clustering further revealed marked differences in miRNA expression among the four age stages, with Y5 showing the greatest divergence from the earlier stages (Figure 5A–C).

To characterize the highly expressed DEMIs, the 20 miRNAs with the highest mean TPM values across the four age stages were selected for expression profiling. These miRNAs exhibited diverse expression patterns, and several belonged to conserved miRNA families associated with plant growth and development, including miR156, miR159, miR166, miR167, miR168, miR171, and miR396 (Figure 5D).

### 3.4. Temporal Expression Patterns of Differentially Expressed miRNAs

To characterize coordinated age-related changes in miRNA expression, the 311 DEMIs were subjected to temporal clustering across Y0, Y1, Y3, and Y5. Twenty expression profiles were identified, of which Profiles 9, 0, 16, and 13 were statistically significant and together contained 210 DEMIs, accounting for 67.52% of the total (Figure 6). Profile 0, comprising 33 DEMIs, showed a continuous decline with increasing shoot age, whereas Profile 9, containing 121 DEMIs, remained relatively stable from Y0 to Y3 before decreasing markedly in Y5. Profiles 13 and 16, containing 24 and 32 DEMIs, respectively, exhibited transient increases followed by declines; Profile 13 peaked in Y1, while Profile 16 maintained relatively high expression in Y1 and Y3. Despite their distinct early-stage trajectories, all four major profiles showed reduced expression in Y5, indicating extensive remodeling of the miRNA expression landscape in the oldest shoots. Fifteen of the 20 most abundant miRNAs were assigned to these significant profiles, including members of the miR156, miR159, miR166, miR167, miR168, miR171, miR319, and miR396 families, demonstrating coordinated but distinct expression dynamics in different age stages.

### 3.5. GO Enrichment and KEGG Pathway Analyses of Predicted Targets of DEMIs

The predicted targets of DEMIs in the four significant expression profiles were subjected to GO and KEGG analyses. GO analysis assigned the targets to 38 functional categories, predominantly including cellular and metabolic processes, biological regulation, response to stimulus, binding, and catalytic activity (Figure 7A). Profiles 9 and 16 showed broader functional distributions than Profiles 0 and 13.

KEGG analysis indicated that the predicted targets were mainly associated with secondary metabolite biosynthesis, starch and sucrose metabolism, fatty acid metabolism, and plant hormone signal transduction (Figure 7B). Starch and sucrose metabolism and the biosynthesis of various plant secondary metabolites were represented across the examined age stages, whereas plant hormone signal transduction and the plant MAPK signaling pathway were particularly prominent in Y3. These results suggested that the predicted targets of age-associated DEMIs may be involved in diverse metabolic and signaling processes in *C. goeringii*.

### 3.6. qRT-PCR Validation of Candidate miRNAs

To evaluate the reliability of the small RNA sequencing data, nine DEMIs were selected for qRT-PCR analysis using *U6* as the internal reference. The qRT-PCR results generally agreed with the expression patterns obtained from small RNA sequencing, although some variation in expression magnitude was observed among individual stages (Figure 7C). Overall, the qRT-PCR analysis supported the reliability of the sequencing-based miRNA expression profiles.

### 3.7. Predicted miRNA–Target Associations Related to Carbohydrate Metabolism and Hormone Signaling

Based on functional analyses of predicted targets, starch and sucrose metabolism and plant hormone signal transduction were further examined because of their close relevance to the age-dependent changes in carbohydrate and hormone contents observed in *C. goeringii*. A number of DEMIs were predicted to target genes involved in sucrose, glucose, trehalose, and starch metabolism (Figure 8). Representative predicted associations included miR1432-x/miR4993-z–*IVR1*, miR163-y–*CIN3*, and novel-m0141-5p–*SUS3*, which were associated with sucrose metabolism, as well as several miRNAs predicted to target *FRK*, *HXK*, and *PGM* family genes involved in hexose metabolism. Multiple predicted targets encoding trehalose-6-phosphate synthases, β-amylases, and α-amylases were also identified. Many of the corresponding miRNAs showed relatively high abundance at Y0 or Y1 and decreased at later age stages, whereas others exhibited transient or stage-specific expression patterns.

Predicted miRNA–target associations were also identified in several major branches of plant hormone signaling, including auxin, cytokinin, gibberellin, abscisic acid, brassinosteroid, and jasmonate signaling (Figure 9). These associations involved key signaling components such as *ARF*, *IAA*, *LAX*, *GH3*, *HK*, *PYL/PYR*, *PP2C*, *SnRK2*, *ABF*, *BRI1*, *BSK*, *BKI1*, *MYC2*, and *JAZ*. For example, several DEMIs were predicted to target auxin-related *ARF* genes, whereas miR4993-z, miR8752-z, and miR7553-z were predicted to associate with components of brassinosteroid signaling. In the jasmonate pathway, several miRNAs were predicted to target *MYC2* or *JAZ* genes and generally showed higher expression at the earlier age stages. Together, these predicted associations provide candidate links between age-dependent miRNA expression and the observed changes in carbohydrate metabolism and hormone signaling.

## 4. Discussion

*C. goeringii* has a relatively long vegetative phase, and the acquisition of flowering competence is closely associated with developmental age. Previous studies have mainly focused on flowering regulation [4,5,6], mutation breeding [29], and stress responses [30], whereas age-dependent changes in flowering competence remain poorly understood. In the present study, floral bud formation showed a clear age-dependent pattern, increasing from Y1 to a maximum in Y2 and subsequently declining, with no floral buds observed in Y0, Y4, or Y5. This non-linear pattern suggests that flowering competence in *C. goeringii* is confined to a specific developmental window rather than increasing continuously with shoot age. Such age-dependent behavior is particularly relevant in perennial plants, in which developmental age may be associated with the physiological status of individual shoots or meristems rather than simply reflecting chronological progression [7,31]. Consistent with the changes in floral bud formation, carbohydrates and endogenous hormones also varied markedly among age stages, indicating substantial physiological remodeling during the establishment and subsequent decline of flowering competence.

Carbohydrates provide both metabolic resources for plant growth and reproductive development and signaling cues that contribute to developmental regulation [7,10]. In the present study, soluble sugar and sucrose contents generally increased with shoot age; glucose reached a relatively high level in Y3 before declining, whereas starch continued to accumulate through Y5. Although soluble sugars have been associated with floral bud differentiation in previous studies [32,33], carbohydrate profiles may vary according to the developmental status and function of individual shoots. In orchids, pseudobulbs serve as important organs for carbon storage and remobilization during subsequent shoot and inflorescence development [34,35]. Thus, the carbohydrate patterns observed among Y0–Y5 may reflect differences in carbon storage and utilization during shoot aging, together with changes in hormonal status and developmental competence. Recent evidence that sugar signaling can directly influence shoot apical meristem function further supports a role for carbon status in developmental timing [10,36]. Endogenous hormones also displayed contrasting age-dependent trends, with IAA, ABA, and BR generally increasing, ZR and MeJA decreasing, and GA remaining comparatively stable. Considering the complex and often context-dependent roles of individual hormones in flowering and development [11], these patterns are more consistent with an age-associated reorganization of hormonal balance than with a simple promotive or inhibitory effect of any single hormone. Recent work showing that cytokinin and GA interact with conserved age-related pathways during vegetative phase change provides additional support for such coordinated regulation [37].

Small RNA sequencing further revealed pronounced age-associated differences in miRNA expression in *C. goeringii*. A total of 311 differentially expressed miRNAs (DEMIs) were identified among Y0, Y1, Y3, and Y5, with comparisons involving Y5 showing the largest numbers of DEMIs and a predominance of downregulation. Temporal expression analysis further showed that Profiles 9, 0, 16, and 13 together contained 210 DEMIs, accounting for 67.52% of the total, and all four significant profiles were characterized by reduced expression in Y5. These patterns indicate extensive remodeling of the miRNA expression landscape with increasing shoot age. Among the identified DEMIs, several developmentally important miRNA families, including miR156, miR159, miR319, and miR396, were particularly noteworthy [15,16,17,18]. The miR156–SPL module is one of the best-characterized components of the plant age pathway [7]. Recent studies in perennial grapevine and lily have further supported the involvement of this module in age-related developmental transitions and flowering [2,38]. In the present study, miR156-x was not only among the relatively abundant DEMIs but also exhibited a significant age-related expression pattern, while members of the miR159, miR319, and miR396 families showed distinct age-responsive dynamics. Taken together, these observations suggest that age-related developmental regulation in *C. goeringii* may involve multiple miRNA modules centered on, but not limited to, miR156. This interpretation is also consistent with recent reviews highlighting the roles of conserved miRNAs and their target transcription factors in developmental timing and hormone-related regulation [39].

Functional analysis of the predicted targets of DEMIs suggested potential links between age-associated changes in miRNA expression and the physiological responses observed in *C. goeringii*. KEGG analysis highlighted starch and sucrose metabolism across different age stages, and this pathway has previously been implicated in floral induction and flower development in species such as apple and loquat [40,41,42]. In the present study, several age-responsive miRNAs were predicted to target genes involved in carbohydrate metabolism, including *SUS, IVR/CIN, FRK, HXK, PGM, TPS, BAM*, and *AMY*. These predicted associations are consistent with the observed age-dependent changes in soluble sugar, sucrose, glucose, and starch contents. Previous studies have also shown that sugar availability can influence miR156 expression [43], while more recent work has further linked sugar signaling with meristem development and developmental timing [10]. In parallel, predicted targets were widely distributed across auxin, cytokinin, GA, ABA, BR, and jasmonate signaling pathways and included key components such as *ARF, IAA, GH3, HK, PP2C, SnRK2, BRI1, MYC2*, and *JAZ*. This broad representation of hormone-related targets is consistent with the age-associated changes in endogenous hormone contents detected in this study. Given the extensive crosstalk between hormone signaling and age-related developmental pathways, together with the recognized role of miRNAs as post-transcriptional regulators linking developmental programs with hormonal responses [39], carbohydrate metabolism and hormone signaling may represent two major functional interfaces connecting age-related physiological remodeling with miRNA expression changes in *C. goeringii*.

Taken together, the present study reveals a dynamic age-dependent pattern in flowering competence in *C. goeringii*, characterized by its establishment, enhancement, subsequent decline, and eventual loss with increasing shoot age. These changes were accompanied by systematic shifts in carbohydrate status, endogenous hormone levels, and miRNA expression profiles. The age-responsive patterns of conserved development-related miRNAs, including miR156, miR159, miR319, and miR396, together with the enrichment of predicted DEMI targets in starch and sucrose metabolism and plant hormone signal transduction, suggest that age-dependent flowering in *C. goeringii* may involve coordination among developmental age signals, metabolic status, and hormonal balance. By linking age-related flowering phenotypes with physiological changes and small RNA profiles, this study provides candidate miRNAs and functional pathways for further elucidating age-related developmental and flowering regulation in perennial *Cymbidium* orchids.

## 5. Conclusions

Age-dependent changes in flowering competence were observed in *C. goeringii*, with floral bud formation increasing from Y1 to Y2 and subsequently declining until it was absent in Y4 and Y5. These changes were accompanied by distinct alterations in carbohydrate contents, endogenous hormone levels, and miRNA expression profiles. Small RNA sequencing identified 311 differentially expressed miRNAs, among which several conserved development-related miRNAs, including miR156, miR159, miR319, and miR396, showed pronounced age-associated expression patterns. Functional analysis of predicted targets further highlighted starch and sucrose metabolism and plant hormone signal transduction as major pathways associated with age-dependent developmental changes. Collectively, these findings provide an integrated physiological and small RNA framework for understanding age-dependent flowering transition in *C. goeringii* and identify candidate miRNAs and pathways for further investigation of flowering regulation in perennial orchids.

## Figures and Tables

**Figure 1:**
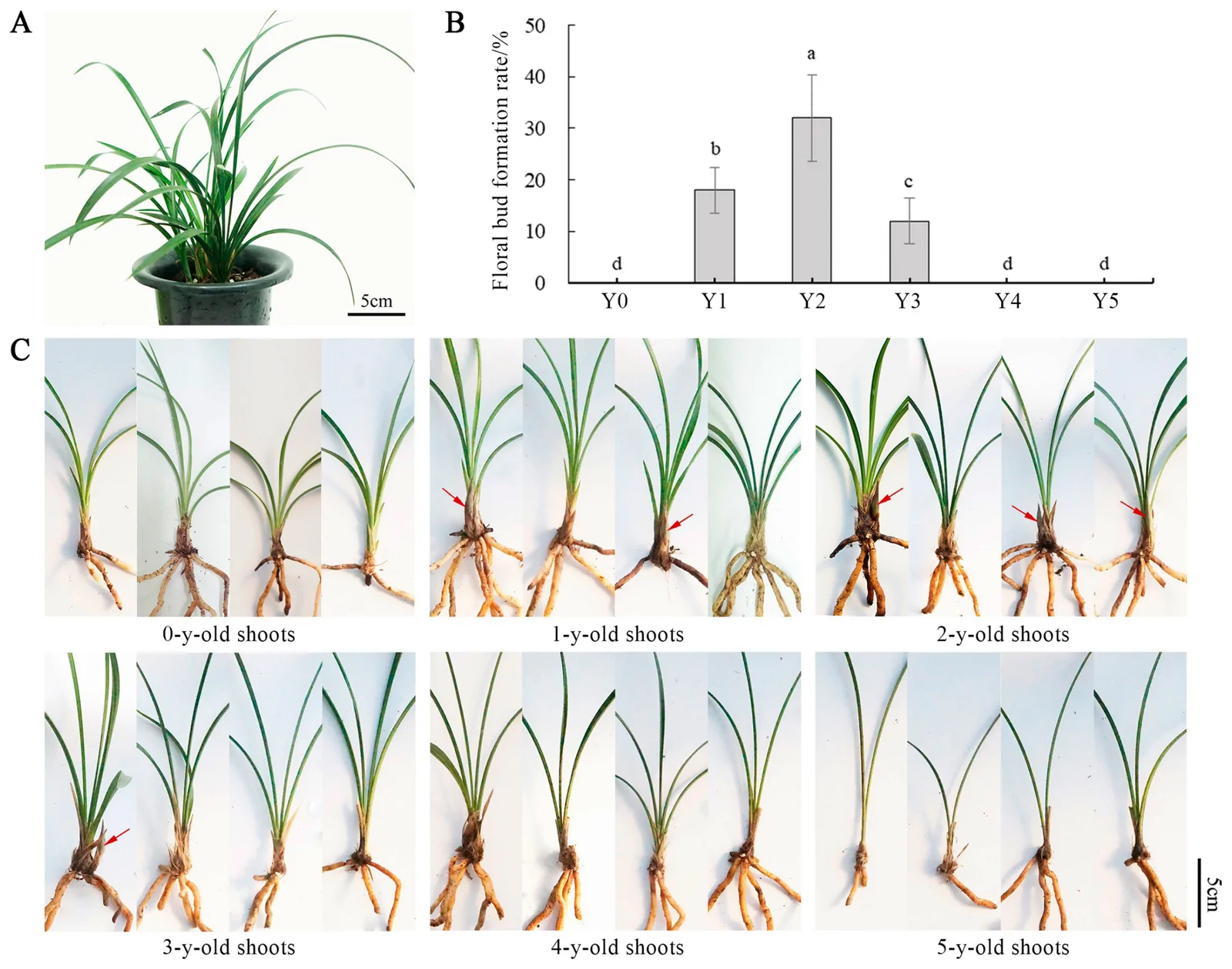
Age-dependent floral bud formation in *Cymbidium goeringii* ‘Songmei’. (**A**) Growth habit of shoots in different age stages cultivated together in the same pot; (**B**) floral bud formation rates of shoots in different age stages. Y0 denotes current-year shoots, whereas Y1–Y5 denote one- to five-year-old shoots, respectively; (**C**) representative morphology of Y0–Y5 shoots after removal of the cultivation substrate. Red arrows indicate floral buds formed at the bases of the pseudobulbs. The error bars indicate the standard deviation. Different lowercase letters above the bars indicate significant differences among age stages at *p* < 0.05.

**Figure 2:**
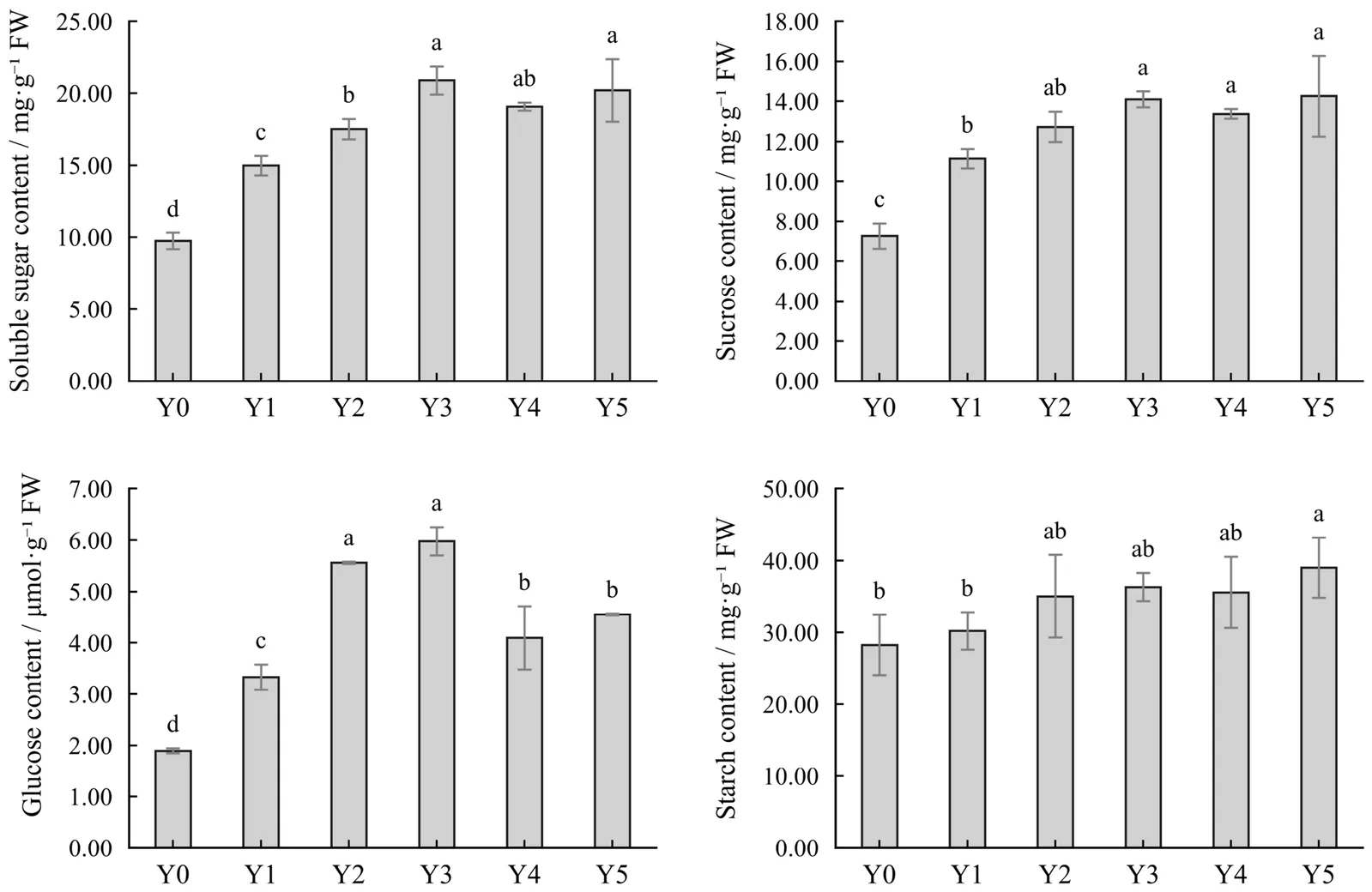
Age-dependent changes in carbohydrate contents in the leaves of *C*. *goeringii* ‘Songmei’. The error bars indicate the standard deviation. Different lowercase letters above the bars indicate significant differences among age stages at *p* < 0.05. FW, fresh weight.

**Figure 3:**
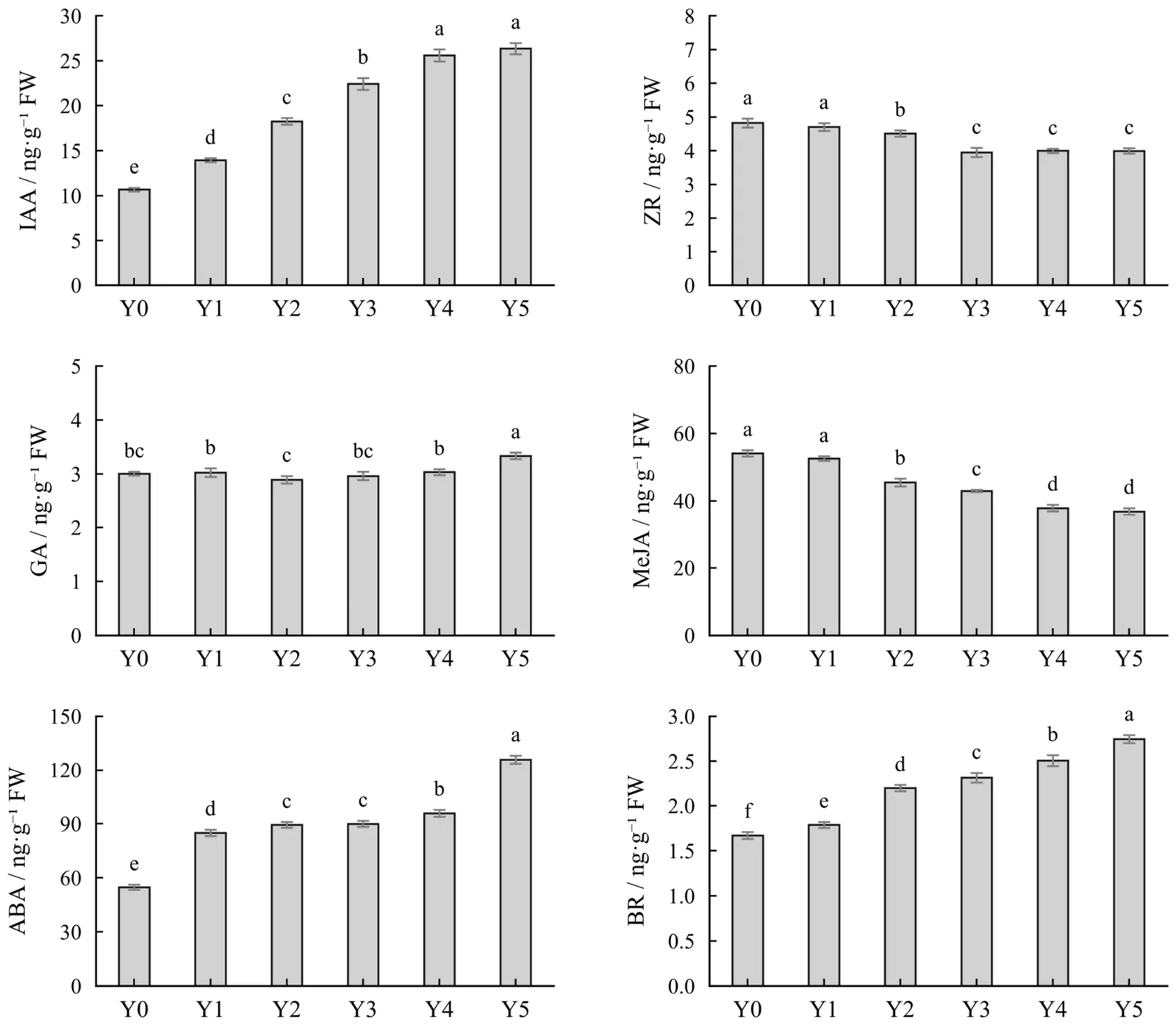
Age-dependent changes in endogenous hormone contents in the leaves of *C. goeringii* ‘Songmei’. The error bars indicate the standard deviation. Different lowercase letters indicate significant differences among different age stages at *p* < 0.05.

**Figure 4:**
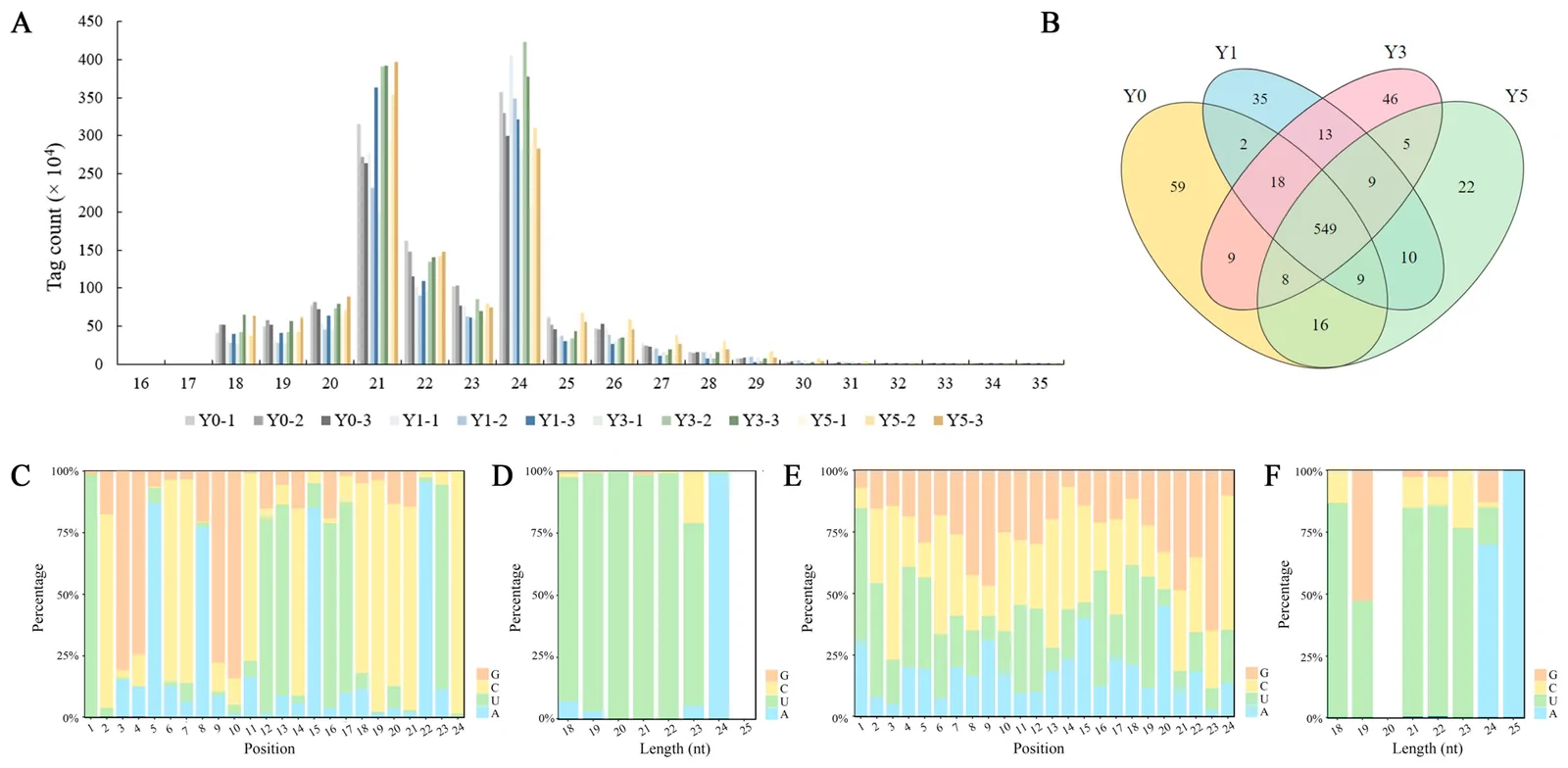
Overview of small RNA sequencing and miRNA identification in *C. goeringii* ‘Songmei’. (**A**) Length distribution of small RNA tags in the 12 libraries constructed from Y0, Y1, Y3, and Y5 shoots. (**B**) Venn diagram showing shared and stage-specific miRNAs among the four age stages; the numbers indicate the identified miRNAs in each overlapping or stage-specific region. (**C**) Nucleotide preference at each position of known miRNAs. (**D**) 5′-terminal nucleotide preference of known miRNAs of different lengths. (**E**) Nucleotide preference at each position of putative novel miRNAs. (**F**) 5′-terminal nucleotide preference of putative novel miRNAs of different lengths.

**Figure 5:**
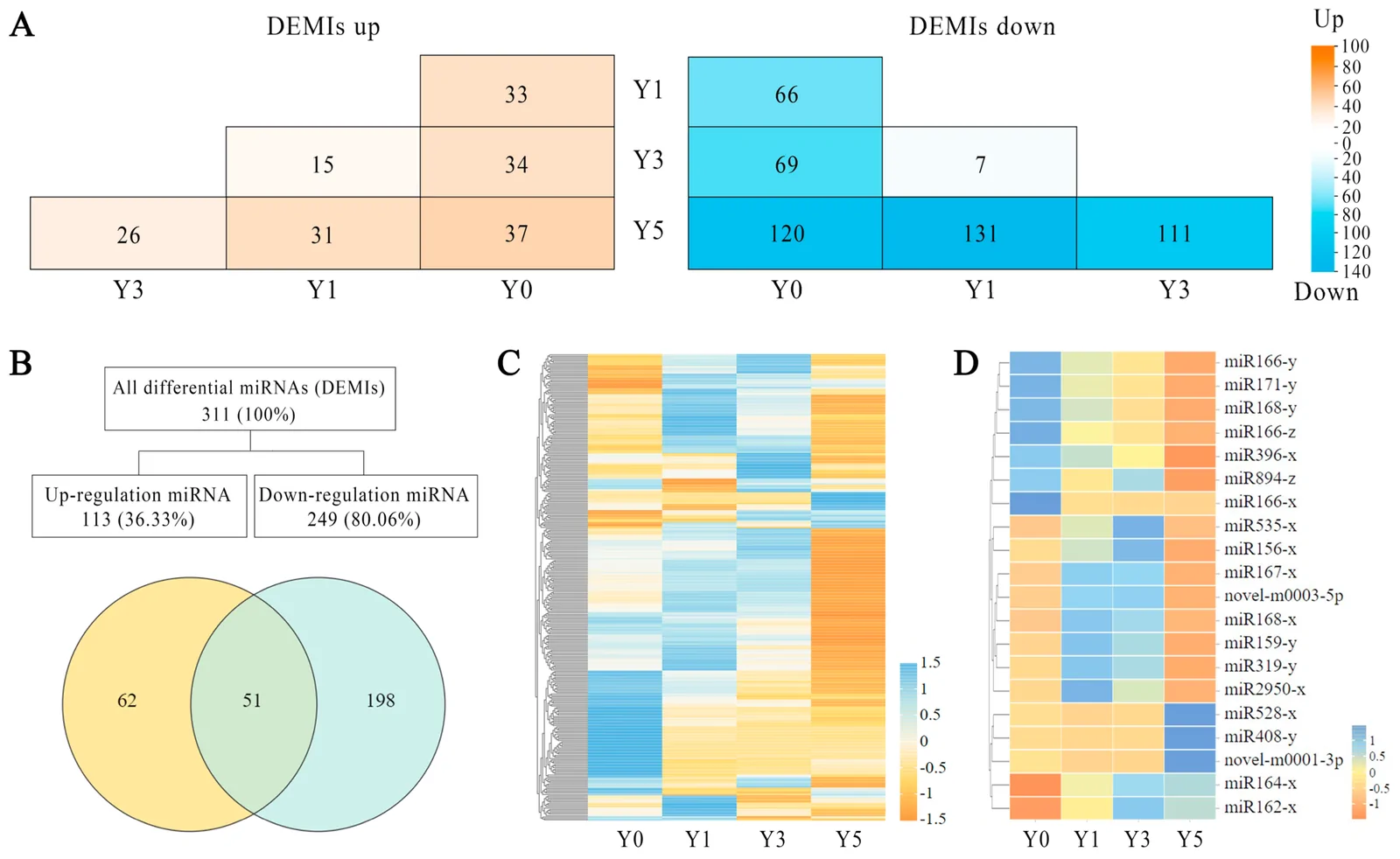
Differential expression and abundance profiles of miRNAs in *C. goeringii* ‘Songmei’ in different age stages. (**A**) Numbers of upregulated and downregulated differentially expressed miRNAs (DEMIs) identified in the six pairwise comparisons among Y0, Y1, Y3, and Y5. (**B**) Summary and overlap of DEMIs showing upregulation or downregulation in at least one comparison; 51 miRNAs exhibited opposite expression trends among different comparisons. (**C**) Hierarchical clustering of the 311 DEMIs based on normalized TPM values. (**D**) Expression profiles of the 20 miRNAs with the highest mean TPM values across the four age stages.

**Figure 6:**
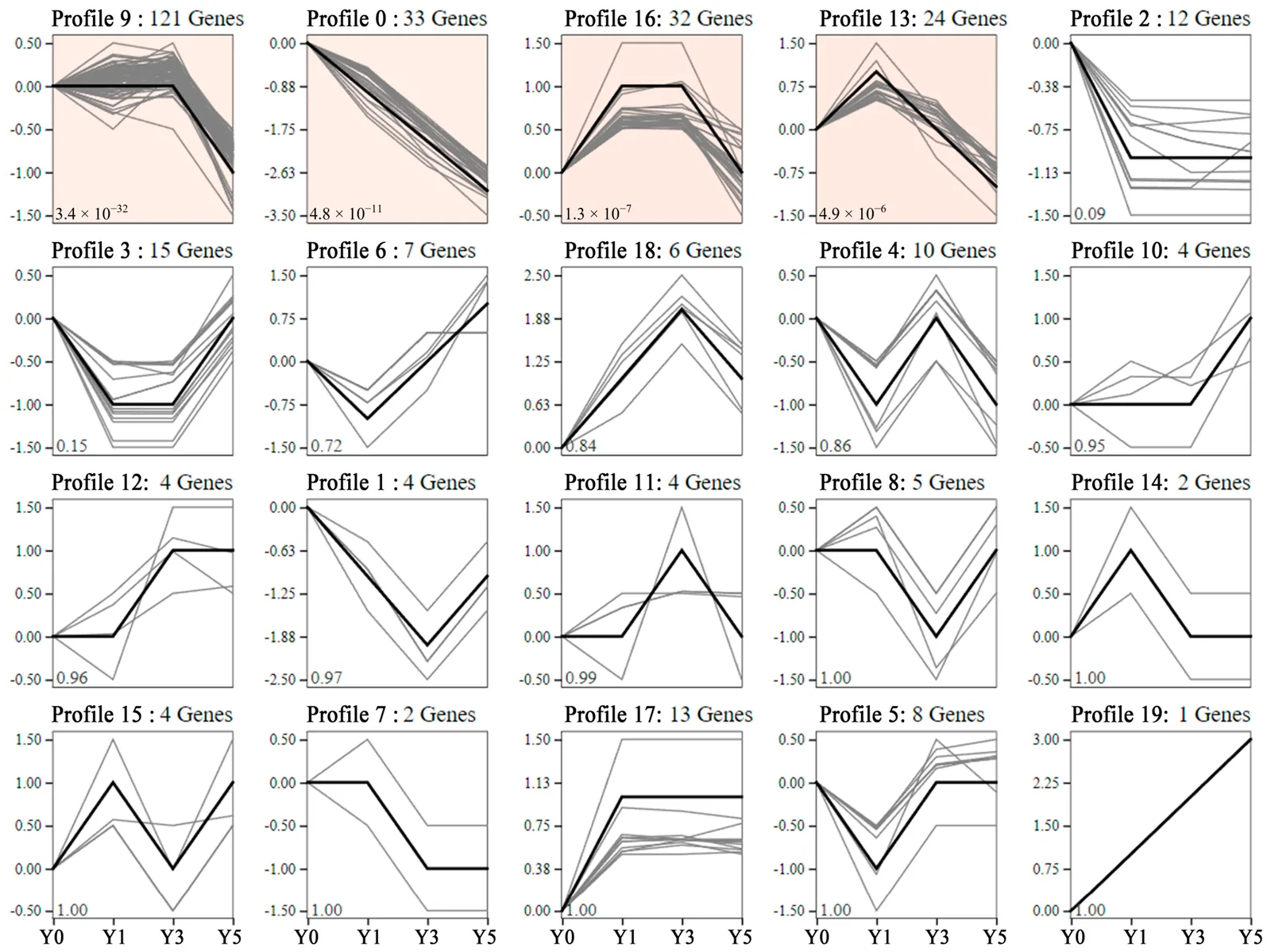
Temporal expression trend analysis of differentially expressed miRNAs in *C. goeringii* ‘Songmei’. The 311 differentially expressed miRNAs (DEMIs) were classified into 20 temporal expression profiles based on their normalized expression patterns in Y0, Y1, Y3, and Y5. Gray lines represent the expression patterns of individual DEMIs, whereas black lines indicate the model expression trend of each profile. The four statistically significant profiles (Profiles 9, 0, 16, and 13) are highlighted with pale pink backgrounds. The number of miRNAs assigned to each profile and the corresponding *p*-value are shown in each panel.

**Figure 7:**
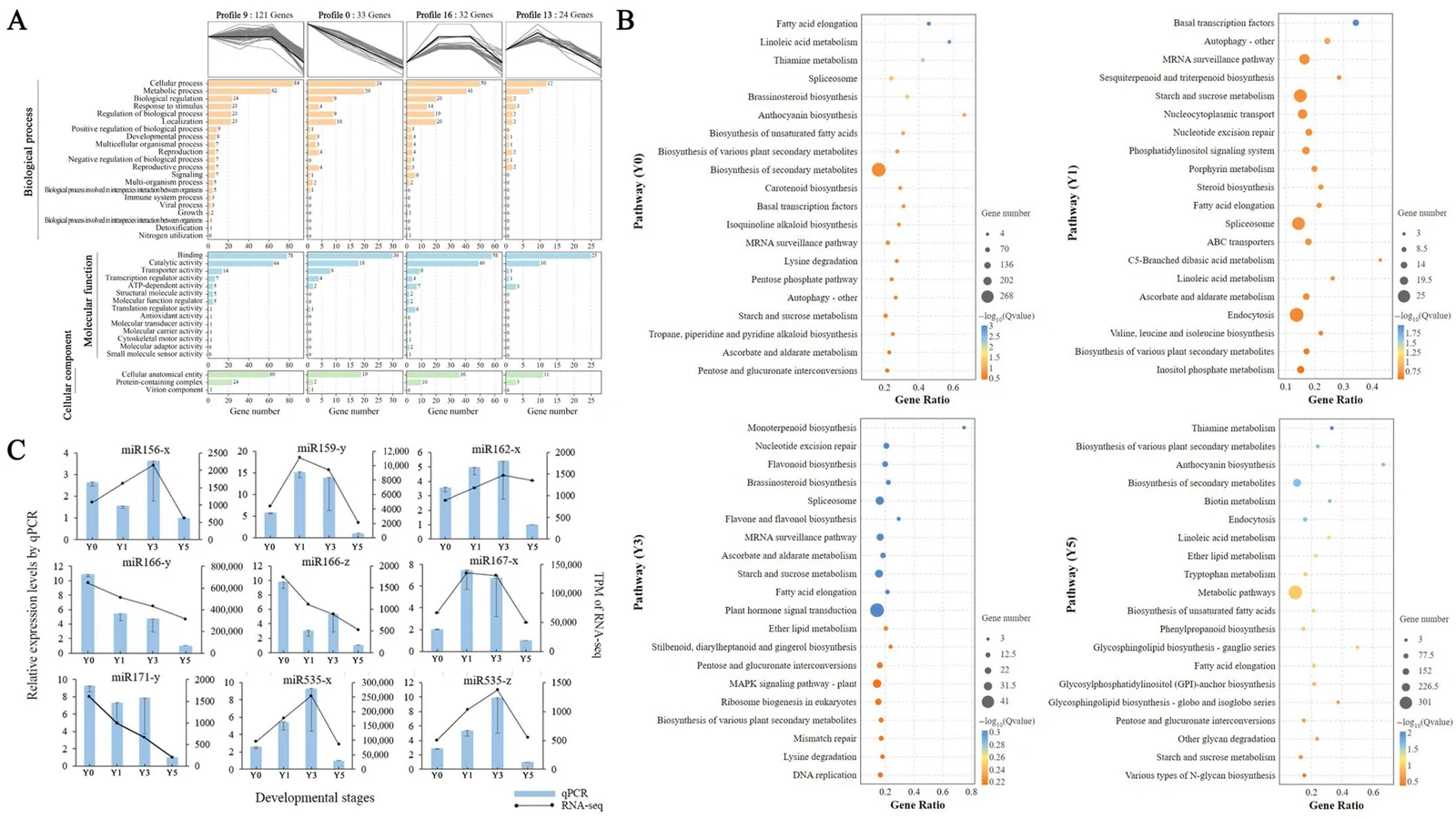
Functional analysis of predicted targets of differentially expressed miRNAs and qRT-PCR validation in *C. goeringii* ‘Songmei’. (**A**) GO functional classification of the predicted targets of DEMIs assigned to the four significant temporal expression profiles. The expression trends of Profiles 9, 0, 16, and 13 are shown above the corresponding GO distributions. Bars indicate the numbers of predicted target genes assigned to biological process, molecular function, and cellular component categories. (**B**) KEGG pathway enrichment analysis of the predicted targets associated with Y0, Y1, Y3, and Y5. The 20 most significantly enriched pathways are shown for each age stage. Bubble size represents the number of predicted target genes, bubble color indicates −log_10_(Q value), and the horizontal axis represents the gene ratio. (**C**) qRT-PCR validation of nine selected DEMIs. Bars represent relative expression levels determined by qRT-PCR using *U6* as the internal reference, whereas black lines represent TPM values obtained from small RNA sequencing.

**Figure 8:**
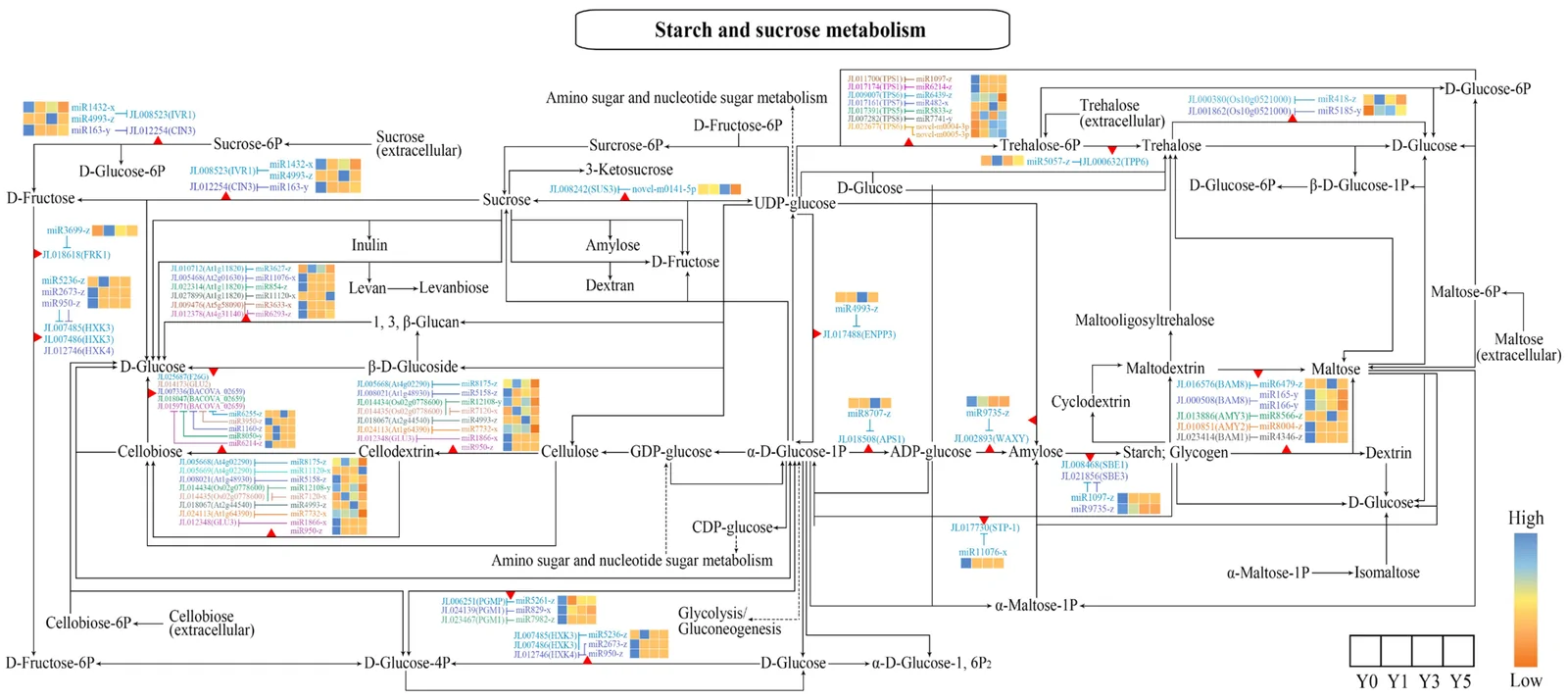
Predicted miRNA–target associations related to starch and sucrose metabolism in *C. goeringii* ‘Songmei’. Predicted target genes of DEMIs were mapped to the starch and sucrose metabolism pathway. Representative genes involved in sucrose, glucose, cellobiose, trehalose, and starch metabolism and their corresponding miRNAs are indicated at the relevant pathway positions. Red triangles mark the positions of predicted target genes in the pathway. Heatmaps show the relative abundance of the corresponding miRNAs in Y0, Y1, Y3, and Y5, with colors ranging from low (orange) to high (blue) expression.

**Figure 9:**
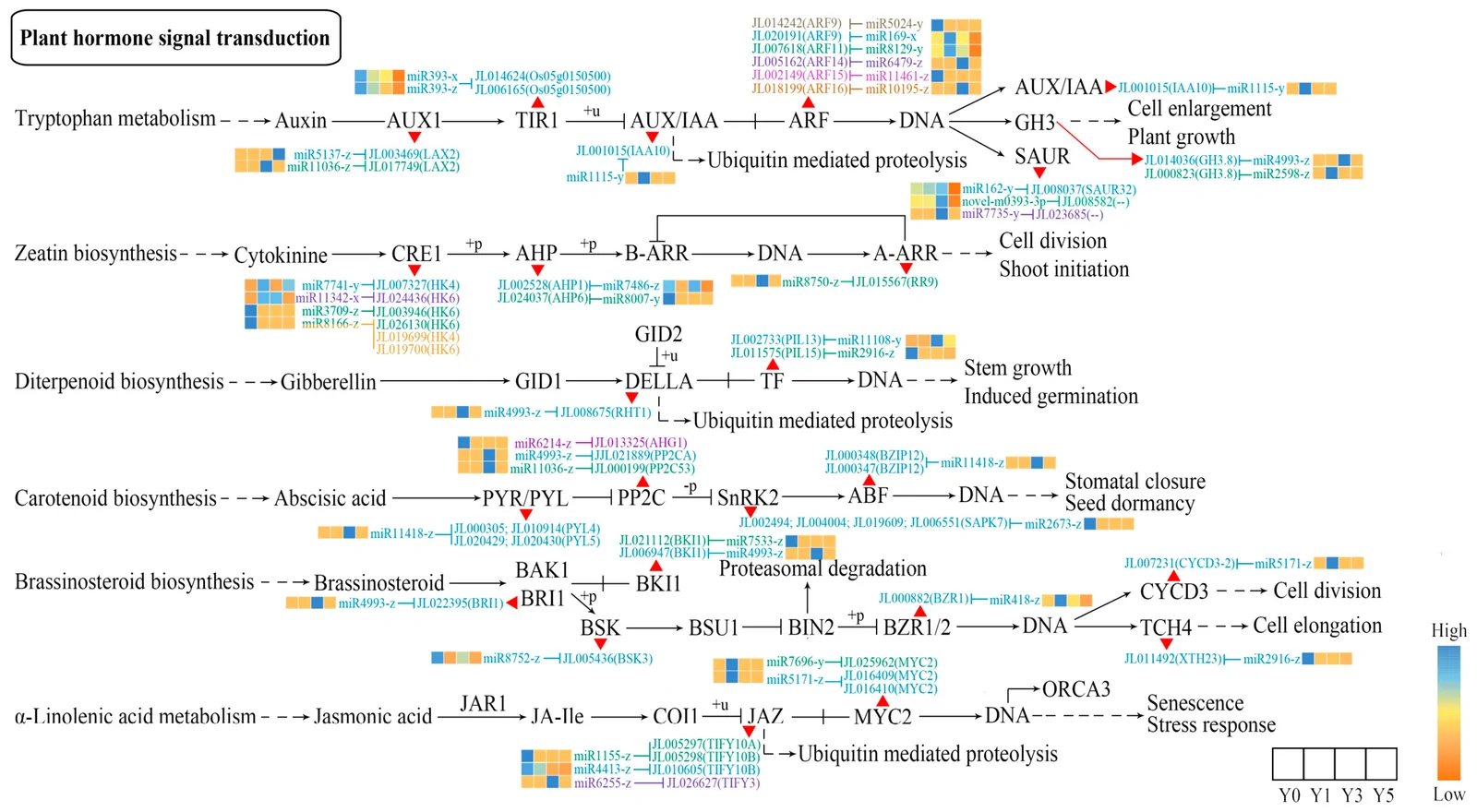
Predicted miRNA–target associations related to plant hormone signal transduction in *C. goeringii* ‘Songmei’. Predicted targets of DEMIs were mapped to the auxin, cytokinin, gibberellin, abscisic acid, brassinosteroid, and jasmonate signaling pathways. Representative predicted miRNA–target associations are indicated at the corresponding signaling components, and heatmaps show the relative abundance of the associated miRNAs in Y0, Y1, Y3, and Y5.

## Data Availability

The data presented in this study are openly available in National Genomics Data Center (NGDC) at https://ngdc.cncb.ac.cn, reference number PRJCA071581.

## Supplementary Materials

The following supporting information can be downloaded at: https://www.mdpi.com/article/10.3390/plants15192899/s1. Table S1: Primer sequences used for miRNA 1st strand cDNA synthesis by stem-loop.
